# A National Population Cohort Study Showed That Exposure to General Anesthesia in Early Childhood Is Associated with an Increase in the Risk of Developmental Delay

**DOI:** 10.3390/children8100840

**Published:** 2021-09-24

**Authors:** Ya-Ling Yang, Liang-Jen Wang, Jung-Chan Chang, Shu-Chen Ho, Ho-Chang Kuo

**Affiliations:** 1 Department of Anesthesiology, Kaohsiung Chang Gung Memorial Hospital and Chang Gung University College of Medicine, Kaohsiung 883, Taiwan; inr453@cgmh.org.tw; 2 Department of Child and Adolescent Psychiatry, Kaohsiung Chang Gung Memorial Hospital, Kaohsiung 833, Taiwan; wangliangjen@gmail.com; 3 Department of Data Science and Analytics, I-Shou University, Kaohsiung 840, Taiwan; jcchang@isu.edu.tw; 4 Department of Public Health, College of Health Sciences, Kaohsiung Medical University, Kaohsiung 807, Taiwan; cho0702@gmail.com; 5 Department of Pediatrics, Kaohsiung Chang Gung Memorial Hospital and Chang Gung University College of Medicine, Kaohsiung 833, Taiwan; 6 Kawasaki Disease Center, Kaohsiung Chang Gung Memorial Hospital, Kaohsiung 833, Taiwan

**Keywords:** general anesthesia, developmental delay, developmental dyslexia

## Abstract

This study investigated the relationship between exposure to general anesthesia (GA) and the risk of cognitive and mental disorders. This study has thus investigated the relationships between exposure to GA before the age of 3 and subsequent cognitive and mental disorders in a national-wide research sample. We obtained our subjects from the National Health Insurance Research Database (NHIRD) of Taiwan, which was based on the International Classification of Diseases, Ninth Revision, Clinical Modification (ICD-9-CM). Children in the hospital aged less than 3 years old were included if there was GA exposure or not during the period of year 1997 to 2008. Cox proportional hazard regression models adjusted for potential confounding factors were used to estimate the relative magnitude of the risk associated with GA exposure. The cohort contained 2261 subjects with GA and 4522 children without GA as a comparison group. GA exposure group had a higher rate of developmental delay than in the without GA group (hazard ratio 1.46, *p* < 0.0001). There was no significant difference in the overall incidence of ADHD, autism and intellectual disability between the GA-exposed group and the comparison cohort. In conclusion, this study reported that children exposed to GA early before the age of three had a small association with increased risk of development delay thereafter.

## 1. Introduction

The medical community is concerned that exposure to certain types of inhaled anesthetics in the early life of animals can enhance immature neuron apoptosis [1], and that such animals might also develop degenerative changes leading to neurocognitive dysfunction [2] and memory [3]. There is increasing evidence that type A gamma-amino butyric acid (GABAA) receptors, which is excitatory rather than inhibitory in the immature neurons, are the main target of sevoflurane. Moreover, sevoflurane is the main inhalation anesthetic used in pediatric surgery [2,4] due to its pleasant odor [5], and it also exerts a protecting effect against bronchoconstriction [6]. The excitotoxic effects of GABAA receptors are related to the increase in neuronal apoptosis after exposure to sevoflurane in animals, which might contribute to sevoflurane-induced subsequent cognitive dysfunction [2]. Besides, it also interferes with dendritic cells development and synaptogenesis, potentially causing cognitive impairment [7]. These pre-clinical literatures have shown changes in neuronal apoptosis that may lead to post-anesthesia cognitive dysfunction [8,9,10]. In addition, sevoflurane can also manipulate memory genes and induce cognitive impairment in aged rodents [11]. More importantly, whether these biological findings of neuronal damage in animal studies will occur in children has become an important issue in the daily anesthesia practice of children. Up to now, although there are many different related studies, the relationships between early childhood exposure to general anesthesia (GA) and the risk of developing attention-deficit/hyperactivity disorder (ADHD) disorders are still under debate [12,13]. However, meta-analysis showed that among children undergoing anesthesia/surgery in early childhood, especially children exposed to anesthesia for multiple times, the risk of adverse neurodevelopmental outcomes was moderately increased [14,15].

Thus, we conduct this nationwide cohort study of children under the age of 3, and investigated the relationships between their GA exposure and subsequent cognitive and mental disorders.

## 2. Methods

As stated in our previous report [16], our research subjects were selected from the National Health Insurance Research Database (NHIRD) of Taiwan, which is based on the International Classification of Diseases, Ninth Revision, Clinical Modification (ICD-9-CM). The National Health Insurance plan provides compulsory universal health insurance for 99% of Taiwan’s 23.74 million residents [17]. Previous studies have described the details of the NHIRD, which consists of a representative database of 1,000,000 randomly sampled subjects and their medical information, including inpatient and outpatient facilities, drug prescriptions, insurance gender, date of birth, date of visit or hospitalization and diagnosis [18,19].

**Figure 1:**
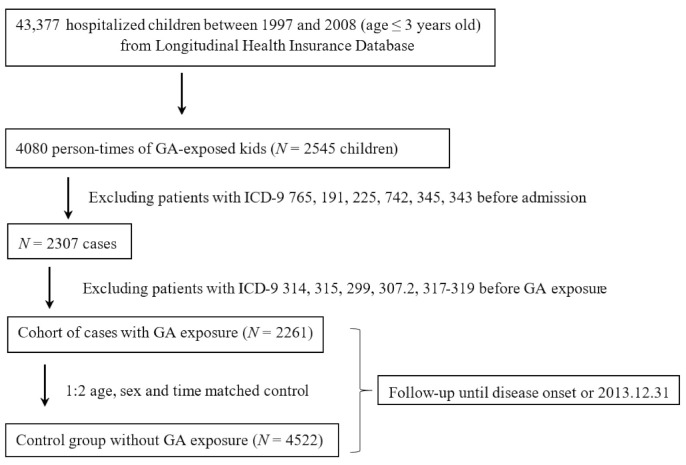
Algorithm for study design and patient selection.

Included in our study sample were children younger than 3 years old who were admitted into hospitals between 1997 and 2008, with or without exposure to general inhalation of anaesthetics. Excluded were those cases with any premature birth (ICD-9-CM 765), brain malignant tumors (ICD-9-CM 191), brain benign tumors (ICD-9-CM 225), other congenital system abnormalities (ICD-9-CM 742), (epilepsy ICD-9-CM 345) and pediatric cerebral palsy (ICD-9-CM 343). Moreover, those who had been diagnosed prior to GA exposure with attention deficit/hyperactivity disorder (ADHD) (ICD-9-CM 314), developmental delay (DD), unspecified (ICD-9-CM 315), autism spectrum disorder (ASD) (Patients with ICD-9-CM 299), tic disorder (ICD-9-CM code 307.2) and intellectual disability (ICD-9-CM 317 to 319) were also removed from this study. According to the management they had received, these patients were divided into two groups: One group was hospitalized patients who had received general anesthesia (GA) and the other group was non-GA-exposed comparison cohorts (matched by age and gender) admitted to the hospital during the same period (±7 days). The comparison-to-study ratio used in this study was 1:2. The Cox proportional hazard model was used to compare between GA-exposed and non-GA-exposed children under 1 year old during the entire follow-up period, the subsequent risk of mental illness including ADHD, developmental delay, unspecified, autism spectrum disorder, tic disorder and intellectual disability. The observation period started on the index day and ended on the day of child’s psychiatric diagnosis or on December 31, 2013. The length of follow-up time was calculated for each patient diagnosed with one of psychiatric diseases. The age and comorbidity of allergic diseases including asthma (ICD-9 493), allergic rhinitis (ICD-9 CM 477) and atopic dermatitis (ICD-9-CM 691) were collected for adjustment. The data collection flow chart is shown in Figure 1. This study using one of the aforementioned databases was exempt from the full review by the Chang Gung Memorial Hospital’s Institutional Re-view Board (IRB No. 102-0364B), as any patient’s ID number in the database is encrypted to protect his or her privacy [20].

### Statistical Analysis

The number of follow-up person-years for each patient was calculated from the date of diagnosis of mental illness to the date of death, or 31 December 2013. The incidence rates were calculated by dividing the number of cases of mentally ill patients by the number of follow-up person-years. Cox proportional hazard regression models adjusted for potential confounders were used to estimate the relative magnitude of risk associated with GA exposure. Participants were divided into two groups: GA exposure or non-GA cohorts. The GA exposure cohort was further divided into mask anesthesia and intubation anesthesia. Hazard ratio (HR) and its 95% confidence interval (CI) were calculated using non-GA patients as a reference. SAS statistical software package (version 9.3; SAS Institute Inc., Cary, NC, USA) was used for analysis. All statistical tests were bidirectional. A *p* value of <0.05 was considered as statistically significant.

## 3. Results

### 3.1. Algorithm for Study Design General Characteristics of the Research Objects

From the NHIRD, 43,377 children below the age of three who were hospitalized between 1997 and 2008 were included in this study cohort. The flowchart of this research is shown in Figure 1. Among them, 2545 subjects and 4080 person times were exposed to GA. After exclusion of 284 (11.16%) subjects (premature birth, brain malignant tumors, brain benign tumors, other congenital system abnormalities, epilepsy and pediatric cerebral palsy, as well as those who had been diagnosed prior to general anaesthesia exposure with ADHD, developmental delay, ASD, tic disorder and intellectual disability), there were 2261 subjects in the study cohort, while 4522 children (in a 1:2 ratio, matched with age, sex and time) made up the comparison cohort (Table 1).

### 3.2. Exposure to General Anesthesia in Early Childhood Has an Association of Increased Risk of Developmental Delay

It is worth noting that the incidence of neurodevelopmental disorders (one of ADHD, developmental delay, ASD, tic disorder and intellectual disability) in the GA-exposed group was higher than that in the non-GA group (11.15% vs. 8.47%, *p* = 0.0004), especially in developmental delay (10.39% vs. 7.36%, *p* < 0.0001). In our previous studies, we have found that allergic rhinitis in childhood may be associated with more symptoms of ADHD [21]; however, allergic diseases do not impair the cognitive development of children [22]. After adjustments for allergic diseases and age, it was still observed that the GA-exposed group had an associated with neurodevelopmental disorders (HR = 1.22; 95% CI = 1.03–1.44), especially, in developmental delay (HR = 1.30; 95% CI = 1.09–1.55) (Table 2). There was no significant difference in the overall incidence of ADHD, autism and intellectual disability between the GA-exposed group and the comparison cohort.

## 4. Discussion

In this study, we reported that after adjustments for allergic diseases and age, a significant correlation between GA exposure and subsequent neurodevelopmental disorders was observed in a nationwide population-based cohort study. Meanwhile, our results indicated that GA exposure in children younger than 3 years old who were admitted into hospitals have no significant association with the later development of ADHD, autism spectrum disorder or intellectual disabilities.

In Tsai et al.’s study, they have observed that children who have been exposed to GA for a long time before the age of 3 are more likely to be diagnosed with ADHD in the future [23]. It must be noted that the within-pair analysis suggested that anesthesia exposure was associated with a higher ADHD score [24] and the later ADHD are associated with prolonged or multiple exposure to anaesthetics [25]. Fortunately, some studies have concluded that children receiving anesthesia in utero [26], in their first 2 years life or later, are not more likely to develop ASD [27]. In addition, an international, multicenter, randomized, controlled equivalence trial has strongly evidenced that an hour of GA in early infancy does not change neurodevelopmental outcome and behavioral measures compared to awake-regional anaesthesia [28]. Our results also showed no significant association with the later development of ADHD, autism spectrum disorder or intellectual disabilities and GA exposure in early life of children. Consistent with the findings of Feng and Kobayashi et al., previous retrospective observational studies also found that children exposed to GA would have an increased risk of DD [29,30]. Compared with healthy siblings who were not exposed to anesthesia, there was no statistical difference in intelligence quotient scores in later childhood [31]. Recently, Robbins et al. have showed that GA for cesarean delivery was not associated with overall DD at two years of age [32]. A previous large-scale cohort study attempted to eliminate differences in socioeconomic and environmental factors, burden of heritable and lifestyle differences affecting children’s development, and showed that children who had GA for surgical procedures before entering primary school were not found to be at increased risk of adverse development outcomes compared with their biological siblings who did not have GA for surgery [33]. Furthermore, Nestor et al. showed no significant differences of psychiatric and neurologic outcomes with any for duration of GA, age at anesthesia, or induction and maintenance agents [34].

However, our study has some limitations that should be mentioned at this point. First, our study was a single-country study, and it still need additional worldwide studies to confirm the effect of GA on developmental delay. Second, it may indicate that children that need surgery itself may develop neurodevelopmental disorders, not necessarily just GA. In the future, additional matched groups are warranted for comparison who have GA only for diagnostic procedures, image studies or removal of foreign bodies such from ear, nose or esophagus, etc. without surgery or minor surgery (such as inguinal hernia, redundant prepuce, phimosis, dental procedure or tongue tie) with major surgery. 

## 5. Conclusions

This report indicates that compared to matched hospitalized pediatric patients, children exposed to GA before the age of 3 may have a mild increase in the risk of developing developmental delays. Delaying general anesthesia for minor operations in this age group may be warranted to a decrease in long-term neurodevelopmental risk.

## Figures and Tables

**Table 1:** General characteristics of the research objects.

Variables	Inpatients without Anesthesia Use (*n* = 4522)		Inpatients with Anesthesia Use (*n* = 2261)		HR (95% CI)	*p* Value
	*n*	%	*n*	%		
Age (means ± SD)	1.90 ± 0.53		1.92 ± 0.60			0.1165
Gender						
Female	1562	34.54	781	34.54		
Male	2960	65.46	1480	65.46		
Follow-up (years, means ± SD)	10.70 ± 3.67		10.48 ± 3.85			0.0230
Neurodevelopmental disorders	383	8.47	252	11.15	1.32 (1.13~1.53)	0.0004
ADHD	139	3.07	67	2.96	0.96 (0.72~1.28)	0.8025
Developmental delay	333	7.36	235	10.39	1.46 (1.22~1.74)	<0.0001
Autism spectrum disorder	43	0.95	18	0.80	0.84 (0.48~1.45)	0.5244
Tic disorders	13	0.29	-	-	-	-
Intellectual disabilities	82	1.81	49	2.17	1.20 (0.74~1.72)	0.3182

Neurodevelopmental disorders indicate patients have one of attention deficit/hyperactivity disorder (ADHD), developmental delay, autism spectrum disorder, tic disorder and intellectual disability; hazard ratio (HR); confidence interval (CI).

**Table 2:** Rates and hazard ratios of neurodevelopmental disorders in exposure to anesthesia before 3 years old.

	Without Anesthesia Use (*n* = 4522)	Anesthesia Use (*n* = 2261)
No. of neurodevelopmental disorders	383	252
Person time (years)	48,402.84	23,699.46
Incidence ^a^	791.28	1063.32
Crude HR (95%CI)	1.00	1.33 (1.13~1.56)
Adjusted HR (95%CI) ^b^	1.00	1.22 (1.03~1.44)
No. of ADHD	139	67
Person time (years)	50,554.95	25,291.04
Incidence ^a^	274.95	264.92
Crude HR (95%CI)	1.00	0.96 (0.71~1.28)
Adjusted HR (95%CI) ^b^	1.00	0.94 (0.69~1.27)
No. of Developmental delay	333	235
Person time (years)	48,713.65	23,804.03
Incidence ^a^	683.59	987.23
Crude HR (95%CI)	1.00	1.46 (1.22~1.74)
Adjusted HR (95%CI) ^b^	1.00	1.30 (1.09~1.55)
No. of Autism spectrum disorder	43	18
Person time (years)	51,148.79	25,580.06
Incidence ^a^	84.07	70.37
Crude HR (95%CI)	1.00	0.84 (0.48~1.45)
Adjusted HR (95%CI) ^b^	1.00	0.72 (0.40~1.31)
No. of Intellectual disabilities	82	49
Person time (years)	50,902.89	25,396.13
Incidence ^a^	161.09	192.94
Crude HR (95%CI)	1.00	1.20 (0.74~1.72)
Adjusted HR (95%CI) ^b^	1.00	1.12 (0.76~1.65)

^a^ per 1000 person-years; ^b^ adjustments for Allergic diseases and age. Neurodevelopmental disorders indicate patients have one of attention deficit/hyperactivity disorder (ADHD), develop-mental delay, autism spectrum disorder, tic disorder and intellectual disability; hazard ratio (HR); confidence interval (CI).

## Data Availability

The datasets generated and analyzed during the current study are not publicly available due to strict ethical regulation of information privacy, but are available from the corresponding author Ho-Chang Kuo on reasonable request.
